# Editorial: Integrative perspectives on the person-context interplay through the lens of temperament

**DOI:** 10.3389/fpsyg.2023.1156267

**Published:** 2023-03-03

**Authors:** Hedwig Teglasi, Samuel P. Putnam, Mirjana Majdandžić

**Affiliations:** ^1^Department of Counseling, Higher Education, and Special Education, University of Maryland, College Park, MD, United States; ^2^Department of Psychology, Bowdoin College, Brunswick, ME, United States; ^3^Research Institute of Child Development and Education, University of Amsterdam, Amsterdam, Netherlands

**Keywords:** temperament, *goodness-of-fit*, person-context dynamics, children, bidirectional, personality, individual differences, emotional development

Temperament is accorded a prominent role in psychology as the biologically-based mechanism by which individuals contribute to their own learning and development, distinct from, but related to, higher-order personality traits involving specific thoughts, values, and conceptions of self, others, and the world (e.g., Henderson and Wachs, 2007). Certain dispositional traits confer vulnerability to adverse outcomes, in part by influencing the “*goodness-of-fit*” between the individual and the surroundings. The role of temperament in the “fit” is complex, involving multiple interrelated layers in the person-context dynamic. Cascade models of development posit that early appearing risk factors are magnified over time because processes that shape children's functioning in one domain progressively influence functioning in other domains (Masten and Cicchetti, 2010). Accordingly, temperamental tendencies that increase risk for maladaptive transactions with the surroundings not only undermine *goodness-of-fit* in the moment, but may also disrupt longer term wellbeing. Temperament plays a role in shaping both overt transactions with the environment and the self-organizing processes of learning from experience, which eventuate in understandings that inform subsequent transactions (e.g., Verron and Teglasi, 2018).

In addressing various aspects of the person-context interplay, the studies in this Research Topic contribute to its overall aim of promoting integrative perspectives on *goodness-of-fit* as involving three layers of transaction between person and context. The person-in-context layer involves moment-to-moment transactions between the individual and the surroundings. Person-as-context encompasses self-organizing processes within the individual, including the interplay of multiple temperamental traits and the cognitive/affective processes that jointly shape what is learned. The assumptions and understandings gained from prior self-organizing processes function as pre-conceptions that influence current responses, referring to person-of-prior-context.

## Person-in-context

Temperament comprises inborn proclivities to respond in certain ways to one's surroundings that, in turn, elicit responses from others, giving rise to reciprocal patterns of transactions. Although temperament is characterized as relatively consistent across time and situations (see Rothbart and Bates, 2006), the *goodness-of-fit* concept implies context as an elicitor of temperamental tendencies.

Certain temperamental tendencies, such as behavioral inhibition, are more salient in some contexts than in others and may be more precisely understood in relation to features of the context. In this Research Topic, Zhou et al. identified three subtypes of fearful temperament that corresponded to situational elicitors (i.e., threat level) and differed in their associations with later anxiety problems, especially in boys. Preschoolers' negative affective reactivity made a unique contribution to social functioning in novel but not in routine contexts, whereas effortful control made a unique contribution to functioning in routine but not novel contexts (Vaughan and Teglasi).

The responses of others (parents, teachers, peers) to the child's individuality are key aspects of the child's context that may enhance or impede the “fit,” moderating and mediating the effects of temperamental risk on developmental outcomes. In this Research Topic, parenting mediated the links between temperament and externalizing problems of children referred with conduct problems (with moderation by sex; Garon-Carrier et al.), and the quality of the teacher-student relationship during preschool moderated the link between temperament and internalizing problems (Susa-Erdogan et al.).

*Goodness-of-fit* is increasingly recognized as the product of bidirectional transactions such as those between broad societal influences and temperamental individuality. Investigated within and across 14 cultures (Pham et al.), bedtime parenting practices (i.e., active and passive sleep-supporting techniques) were related to both cultural context and toddler temperament. The dynamic nature of parent-child exchanges over time is captured in studies that model reciprocal influences longitudinally. Even before a child is born, parents have expectations that, along with the child's actual characteristics, have been found to shape parent-child transactions (Van den Akker et al.). Underscoring long-term bidirectional influences, Tan and Smith found that child negative affectivity influenced maternal expression of negative emotions and that maternal negative expressivity influenced child negative affectivity, but only in specific age periods. Finally, investigating reciprocal relations between child temperament and engagement with contextual stimuli, Fitzpatrick et al. demonstrated that toddler screen media intake predicted subsequent lower effortful control, but not vice versa.

## Person-as-context

Individuals are endowed with multiple temperamental dispositions, and others respond to the whole child, not to isolated traits. For these reasons, there is increasing appreciation of *person-centric* research, the child as context. Using latent profile analysis of teacher-rated child temperament, Martin and Lease found that the configuration of temperamental traits of a particular child (person-as-context) was associated with other children's perceptions of their influence on peers in school and with socially relevant attributes (person-of-prior context), and that temperament profiles associated differently with peer influence depending on the school community (person-in-context).

Though understudied, the role of temperament on *goodness-of-fit* is augmented through its relations with the self-organizing processes by which the individual makes sense of, and learns from, experiential “data.” For example, young children's emotional tendencies were related, over time, to how they processed social information (Davies et al., 2020). In this Research Topic, Zdebik et al. investigated uncertainty intolerance as an aspect of social information processing arising from both temperament and attachment, and found that uncertainty intolerance mediated the relation between behavioral inhibition in childhood and adult generalized anxiety disorder. Over time, self-organizing processes eventuate in pre-conceptions (e.g., cause-effect connections; assumptions, understandings) that subsequently inform transactions and self-organization. Smith et al. considered the multiple interactive influences of cognitive/affective experiences within adolescents, where temperament moderated the effects of appraisal and coping on psychopathology. In these ways, temperament individualizes the “raw data” of experience not only by eliciting responses from others but also by shaping the organization of the subjective world (Teglasi and Epstein, 1998).

## Person-of-prior-context

Pre-conceptions, consolidated through prior self-organizing processes take on a life of their own, influencing subsequent responses and, by extension, *goodness-of-fit*. For example, children's understanding that beliefs drive actions (theory of mind) correlated with the effectiveness of their social behavior (Teglasi et al., 2022). In this Research Topic, the finding that uncertainty intolerance, which shapes information processing, mediated the relation between child temperament and adult outcomes (Zdebik et al.) speaks to the role of prior information processing (person-as-context) on subsequent wellbeing (person-of-prior context). The study of Smith et al. also alludes to the person-of-prior-context perspective because coping and appraisal are products of the synthesis of prior transactions.

To fully unpack the factors influencing *goodness-of-fit* at any particular time in development, it is necessary to consider current transactions (*in context*), the intra-individual dynamic, including self-organization (*as context*), and the impact of prior understandings and temperament on both the transactions and self-organizing processes (*of context*). The studies in this Research Topic support a dynamic conception of *goodness-of-fit* as encompassing an ongoing interplay among temperament, pre-conceptions, context, and self-organizing processes that accommodates the complexities of temperament-informed assessment and interventions across the age span.

## Author contributions

HT drafted the editorial which was informed by discussion among the authors. SP and MM provided feedback. All authors contributed to the article and approved the submitted version.
